# Underneath Images and Robots, Looking Deeper into the Pneumoperitoneum: A Narrative Review

**DOI:** 10.3390/jcm13041080

**Published:** 2024-02-14

**Authors:** Guido Mazzinari, Lucas Rovira, Kim I. Albers-Warlé, Michiel C. Warlé, Pilar Argente-Navarro, Blas Flor, Oscar Diaz-Cambronero

**Affiliations:** 1 Perioperative Medicine Research Group, Health Research Institute la Fe, Avenida Fernando Abril Martorell 106, 46026 Valencia, Spain; argente_marnav@gva.es (P.A.-N.); oscardiazcambronero@gmail.com (O.D.-C.); 2 Department of Anesthesiology, La Fe University Hospital, Avenida Fernando Abril Martorell 106, 46026 Valencia, Spain; 3 Department of Statistics and Operational Research, University of Valencia, Calle Doctor Moliner 50, 46100 Burjassot, Spain; 4 Department of Anesthesiology, Consorcio Hospital General Universitario de Valencia, Av. de les Tres Creus, 2, L’Olivereta, 46014 València, Spain; lucasrovira@gmail.com (L.R.); blasflor@hotmail.com (B.F.); 5 Department of Colorectal Surgery, La Fe University Hospital, Avenida Fernando Abril Martorell 106, 46026 Valencia, Spain; kim.albers@radboudumc.nl; 6 Department of Anesthesiology, Radboud University Medical Centre, 6525 GA Nijmegen, The Netherlands; 7 Departments of Surgery, Radboud University Medical Centre, 6525 GA Nijmegen, The Netherlands; michiel.warle@radboudumc.nl

**Keywords:** pneumoperitoneum, laparoscopic surgery, minimally invasive surgery, intra-abdominal pressure (IAP), peritoneal homeostasis, abdominal compliance, neuromuscular blockade (NMB), perioperative temperature control

## Abstract

Laparoscopy offers numerous advantages over open procedures, minimizing trauma, reducing pain, accelerating recovery, and shortening hospital stays. Despite other technical advancements, pneumoperitoneum insufflation has received little attention, barely evolving since its inception. We explore the impact of pneumoperitoneum on patient outcomes and advocate for a minimally invasive approach that prioritizes peritoneal homeostasis. The nonlinear relationship between intra-abdominal pressure (IAP) and intra-abdominal volume (IAV) is discussed, emphasizing IAP titration to balance physiological effects and surgical workspace. Maintaining IAP below 10 mmHg is generally recommended, but factors such as patient positioning and surgical complexity must be considered. The depth of neuromuscular blockade (NMB) is explored as another variable affecting laparoscopic conditions. While deep NMB appears favorable for surgical stillness, achieving a balance between IAP and NMB depth is crucial. Temperature and humidity management during pneumoperitoneum are crucial for patient safety and optical field quality. Despite the debate over the significance of temperature drop, humidification and the warming of insufflated gas offer benefits in peritoneal homeostasis and visual clarity. In conclusion, there is potential for a paradigm shift in pneumoperitoneum management, with dynamic IAP adjustments and careful control of insufflated gas temperature and humidity to preserve peritoneal homeostasis and improve patient outcomes in minimally invasive surgery.

## 1. Towards the Minimally Invasive Pneumoperitoneum in Laparoscopy

Laparoscopic access has improved surgical results compared to open surgery, and it is now regarded as the gold standard for many indications [1,2,3,4,5]. It induces less direct trauma with smaller incisions. It has been associated with less postoperative pain, less systemic immunological depression, less wound infection, fewer complications, faster bowel recovery, shorter hospital stays, and earlier return to normal activities compared to open surgery [6]. Since its inception [7], apart from the continuous evolution of surgical instruments [8], two major advancements have been implemented. The first is related to image quality enhancement introduced by high definition and 3D depth [9,10], and the second is linked to the accuracy offered by robotic surgical procedures [11,12]. All these technical aids are aimed at facilitating the work of the surgical team and indirectly benefit the patient [13].

Conversely, the insufflation process, another critical component of the laparoscopy technique, has experienced minimal modifications. Its principle is to expand the virtual cavity that resides between the peritoneal membrane layers. However, the peritoneal membrane is far from inert. Its mesothelial cells are flattened squamous cells with epithelial-like microvilli that produce various substances like glycosaminoglycans, phospholipids, proteoglycans, surfactants, and coagulant precursors, forming a protective glycocalyx barrier. This barrier prevents shear stress, infections, and cancer spread, allowing the smooth sliding of serosal surfaces. Moreover, mesothelial cells are connected by tight junctions, integrins, and cadherins, anchored to the actin cytoskeleton, and produce fibronectin, laminin, and collagen, forming the basal membrane. Ultimately, they control fluid and cell transport, inflammation, tissue repair, fibrin deposit lysis, protection against microorganisms, and, potentially, tumor dissemination in the coelomic cavity [14]. Therefore, while gas insufflation in the abdominal cavity, i.e., the pneumoperitoneum, generates a suitable workspace for the surgeon, it can potentially impair the patient’s peritoneal homeostasis by causing reduced peritoneal blood flow, peritoneal acidosis, oxidative stress, mesothelial alterations, and the formation of adhesions [15]. The pneumoperitoneum’s chemical, physical, and biological features may affect clinical outcomes such as postoperative pain, recovery time, and the length of hospital stay [16,17,18]. The injury that occurs with conventional insufflation is due to the variation in three fundamental parameters: the gas psychometric features (i.e., temperature and humidity) and the amount of pressure and gas volume administered. In this narrative review, we will review the mechanism of injury and which implementations can potentially reduce it.

## 2. Pneumoperitoneum Effects and the Pressure–Volume Derivative

Even though the European Association for Endoscopic Surgery suggests adjusting intra-abdominal pressure (IAP) to the minimum level necessary for a sufficient surgical workspace, it is a widespread clinical practice to maintain a constant IAP between 12 and 15 mmHg [19]. This approach is based on the dubious assumption that applying more pressure always allows for more space. Yet, like all bodies with rigid borders, the abdominal cavity cannot expand indefinitely. According to biomechanics laws, materials initially undergo an elastic phase where there is almost a linear relationship between the stress, i.e., the force applied, and strain, i.e., the deformation that they experience. Eventually, a point of maximum stretch (the yield stress) is reached, where additional stress results in only marginal deformation [20,21].

Both animal and human trials have confirmed this nonlinear stress–strain relationship between the administered IAP and the gain in intra-abdominal volume (IAV) during pneumoperitoneum. A recent individual patient meta-analysis of patients undergoing laparoscopic surgery analyzed pressure and volume data recorded at the start of insufflation and showed how a non-linear model fitted the pressure–volume (PV) relationship better than a linear model and how a critical point where the abdominal compliance, i.e., the amount of gained volume for an increment in pressure, decreases can be determined by using the function’s first derivative [22]. Similar results were observed by computed tomography (CT) imaging in a porcine animal model of abdominal insufflation, where gains in IAV were shown to be diminishing [23]. Thus, during pneumoperitoneum, the yield stress point is eventually reached where the IAP–IAV curve comes to a plateau; further increases in pressure past such point result in only marginal gains in volume [24]. Of note, the point occurs in the usual range of IAP used for pneumoperitoneum, generally around 10 mmHg, and it can be located by applying non-linear functions such as logistic or exponential to model abdominal behavior during the insufflation [25]. Acknowledging and responding to this fundamental physical principle is crucial, as elevated IAP is associated with various negative effects. In comparison, intra-abdominal hypertension (IAH), i.e., an abdominal pressure above 12 mmHg, is a well-known and acted upon clinical condition in critically ill patients that increases morbidity and mortality through decreased abdominal organ perfusion and increased pressure transmitted to other body organs [26,27,28,29,30,31,32]. Indeed, a raised IAP can displace the diaphragm upward, thus causing a compression of the intrathoracic content that leads to increased transpulmonary pressure and decreased cardiac venous return [33]. This pressure transmission is even more important if body positioning has to be taken into account. For instance, increased intracranial and intraocular pressure has been associated with laparoscopic surgery performed in the Trendelenburg position [34,35,36].

Several studies carried out during pneumoperitoneum insufflation confirm that the pressure increment is associated with morphological and physiological changes to the peritoneal microenvironment that are correlated with the duration of such pressure increment during surgery. For instance, IAP was found to be related to the grade of peritoneal morphological changes such as mesothelial cell bulge and basal lamina exposure in a murine model comparing pneumoperitoneum insufflated at different thresholds compared to control animals submitted to general anesthesia only [37]. In a study carried out on patients undergoing laparoscopic cholecystectomy, the electronic microscopy examination of peritoneal tissue highlighted signs of distress such as edema and cell apoptosis [38]. IAP levels are also associated with the increased release of proinflammatory cytokines and decreased reactive oxygen species scavengers, macrophage activity, and glycocalyx extracellular matrix protein synthesis [39,40]. Moreover, high IAP levels can impair organ perfusion [41]. Both hepatic microcirculation measured by laser Doppler and portal venous flow measured by ultrasound were found to be decreased in patients undergoing laparoscopic cholecystectomy [42,43]. More recent data assessed hepatic blood flow indirectly by the indocyanine green plasma disappearance rate (ICG-PDR) and showed how ICG-PDR was higher and, hence, liver perfusion was lower in patients who underwent colorectal laparoscopy with 12 mmHg of IAP compared to those who received 8 mmHg of IAP [44]. ICG was also employed in another study that assessed peritoneal capillary microcirculation by computerized image analysis in patients who underwent robotic colorectal surgery at 8, 12, or 16 mmHg of IAP. Upon intravenous injection of ICG, the time to reach maximal fluorescent intensity (TMFI) and the maximum fluorescent intensity (MFI) were compared among groups, and shorter TFMI and higher MFI were observed in the low IAP group [45]. Furthermore, cells that are under threat or become damaged release damage-associated molecular patterns (DAMPs); these intracellular molecules normally function as danger signals by binding to toll-like receptors on innate immune cells. In the context of (surgical) trauma and tissue damage, high DAMP levels lead to immune dysregulation resulting in immune suppression and increased susceptibility to infections. [46,47,48].

As for clinical outcomes, several meta-analyses have compared the effect of low IAP on perioperative outcomes in patients undergoing laparoscopic cholecystectomy. A Bayesian network meta-analysis comparing low, i.e., 8 mmHg, standard, i.e., 8–16 mmHg, and high, i.e., over 16 mmHg, IAP showed lower pain scores and shoulder pain at postoperative 24 h in the low IAP arm while no differences were observed in other outcomes such as the need for conversion to open surgery, postoperative nausea and vomiting (PONV) or bleeding, and the duration of surgery or hospital stay [49]. A more recent one with a larger sample size confirmed these results with a high overall quality of evidence according to the Grading of Recommendations, Assessment, Development, and Evaluations (GRADE) criteria [50]. Other systematic reviews evaluated the perioperative effect of IAP level during laparoscopy without restricting it to a specific surgical indication. A meta-analysis merging randomized and observational studies compared the effect of low IAP, defined as less than 10 mmHg, with standard IAP, i.e., more than 10 mmHg, during laparoscopy and found lower pain scores in the low IAP ram and no other effect with low quality of evidence [51]. A more recent one that included 85 randomized controlled trials (RCTs) and 7349 subjects compared low (less than 10 mmHg) vs. standard (10 mmHg or higher) IAP during laparoscopy and found that the low IAP arm had a lower incidence of postoperative complications, defined as Clavien–Dindo grade 1 or 2, lower pain scores, lower incidence of PONV, and reduced length of hospital stay without increasing intraoperative adverse events [52]. Accordingly, recent RCTs have linked low IAP during pneumoperitoneum with improved postoperative recovery focusing on the length of hospital stay [53] or postoperative recovery assessed as a patient-reported outcome on a multidimensional score such as the postoperative quality of recovery score (PQRS) [54]. Both RCTs report better outcomes in the low IAP arm.

## 3. Individualizing Pressure and Volume Measures to Increase the Workspace

Several factors collectively influence the determination of the ideal IAP to balance minimal physiological side effects and an optimal surgical field. These factors include the compliance of the abdominal region, the positioning of the patient during surgery, the depth of neuromuscular blockade, the nature of the surgical procedure, and whether it involves the upper or lower abdomen. Additionally, considerations extend to the procedural complexity, with more intricate surgeries requiring precise adjustments to intra-abdominal pressure for improved visibility and control. The surgeon’s experience is a pivotal aspect, as their skill and expertise contribute to adapting and optimizing IAP based on the specific needs of each surgery. Complicating elements such as intra-abdominal fat, bleeding, or adhesions add an extra layer of complexity. These factors must be carefully evaluated, and IAP should be adjusted accordingly to accommodate these challenges and minimize potential complications.

As previously mentioned, the published evidence shows the beneficial effects of low-pressure pneumoperitoneum at an IAP of <10 mmHg for a wide range of surgical procedures [52]. It should be considered that while IAP levels are important for all the reasons we exposed in the previous section, the actual IAV greatly varies between individuals with the same IAP, and it is the variable that ultimately defines the surgical workspace. Mathematical models show that abdominal compliance can show a mostly linear relationship between pressure and volume up to 12 mmHg but stress the large intra-individual variation in volume at the same level of pressure, illustrated by a difference of a whole liter between two patients in the supine position at an insufflation pressure of 10 mmHg [55]. Indeed, the mean insufflated volume for the same pressure can increase by almost a liter with hip flexion or adopting the Trendelenburg position [55]. A previously published study estimated that the required IAV for an adequate surgical view is around 3L and shows how the IAV at different pressures is greatly influenced by body positioning. The optimized IAV was estimated by analyzing the initial pneumoperitoneum insufflation with low flow of 1–2 L m^−1^ and drawing a graph between pressure and volume. This study also assessed which factors can be associated with the optimized IAV by fitting a random linear mixed model assessing the effect of age, gender, body mass index (BMI), number of pregnancies, number of previous laparoscopic or open surgeries, type of surgery (right or left hemicolectomy, rectum, or other surgeries), IAV at 15 mmHg of IAP during the insufflation maneuver, and IAP at volume zero. They found that the variables associated with the optimized IAV were IAV at 15 mmHg and IAP at volume zero [56]. An appealing strategy can be to set this volume as the clinical target and use whatever IAP is needed to achieve it as long as the elastic recoil of the abdominal wall is maintained. Although shifting the focus from pressure to the volume of gas required for maintaining an adequate workspace may appear attractive, it is the pressure level that negatively affects peritoneal blood flow, leading to damage, and, consequently, it should be monitored. For instance, capillary perfusion pressure is estimated to drop to 10 mmHg in the distal segments, providing further support for maintaining a threshold of less than 10 mmHg for a minimally invasive pneumoperitoneum during laparoscopy [57]. A sensible course of action could be individualizing treatment and titrating the right IAP level for each surgical case. This approach allows for the dynamic adjustment of pressure based on the specific requirements of the surgeon, patient, and procedure. In fact, although the overall goal is to conduct the surgery with an IAP below 10 mmHg, it is vital to acknowledge that only the surgeon can assess the adequacy of the surgical working field and decide whether an increase in pressure might be necessary. While utilizing low pressure in laparoscopy offers considerable advantages, prioritizing surgical safety remains paramount. It could very well be the case that in patients with poor abdominal compliance in a difficult clinical scenario, such as pelvic resection, more IAP can be needed, but in general, following the European Association for Endoscopic Surgery guidelines should be the guiding principle.

Indeed, several measures have been suggested to increase surgical workspace besides increasing IAP. For instance, pre-stretching of the abdomen has been proposed to achieve an increased IAV. In a porcine model without neuromuscular blockade (NMB) a stepwise peritoneal insufflation maneuver up to 15 mmHg of IAP increased the subsequent IAV and the anteroposterior abdominal diameter reached upon reinsufflation at each 5 mmHg IAP step as measured by computerized tomography (CT) scan [58]. Pre-stretching by raising IAP at 15 mmHg for 5 min when establishing the pneumoperitoneum was implemented as part of a multifaceted strategy for an individualized lower IAP during laparoscopy that yielded clinical benefits compared to standard pressure pneumoperitoneum [54,59]. A metabolic ischemia–reperfusion protective response has also been postulated in addition to this mechanical effect [60,61]. However, the effects of this technique on the peritoneum microenvironment are still unclear. Other studies carried out in humans reported conflicting results on the actual efficacy of this intervention and its marginal effect and interaction with NMB depth [62,63].

Indeed, the optimal depth of NMB for optimal laparoscopic conditions remains an unresolved issue. During moderate NMB, 75% to 90% of receptors in the neuromuscular junction are blocked with an NMB drug, and one to three train-of-four (TOF) responses using neuromuscular monitoring are obtained. Deep NMB is defined by no TOF responses and quantified by at least one post-tetanic count (PTC) response. A PTC of at least one constitutes a deep NMB [64]. There is an abundance of relatively small trials comparing deep with moderate or no NMB, and comparison is complicated as many trials have also applied different IAPs for both intervention arms. A meta-analysis reported a better visual rating as reported by the operating surgeon with deep NMB compared with moderate NMB [65]. Nonetheless, magnetic resonance imaging (MRI) measurements of pneumoperitoneum volumes showed only slight increases from moderate to deep NMB [66]. The additional perceived improvement of the surgical field by the surgeon may be the result of improved surgical stillness and less sudden abdominal contractions that can improve intraoperative safety, especially when operating close to delicate structures [67]. It can be appealing to resort to a deep NMB, especially since the option of reverting it safely is available with sugammadex [68,69,70]. It should be noted that postoperative residual curarization (PORC) is a well-known clinical condition that has been associated with increased postoperative morbidity [71], and it can be observed even after sugammadex reversal [72]. Given the potential for significant differences in how patients respond and the potential drawbacks of a deep NMB, we recommend that low intra-abdominal pressure (IAP) should be supported with at least moderate NMB and the depth of NMB can be increased as needed.

In other words, surgical teams should carefully adjust intra-abdominal pressure (IAP) during laparoscopic procedures, considering factors like the type and complexity of the surgery, their own experience, and the patient’s specific characteristics. However, it’s essential to recognize that raising IAP does not always result in more space or a better surgical view. Surgeons should be mindful not to compromise safety by pursuing lower pressure at the expense of the patient’s well-being. In summary, IAP is a dynamic parameter that surgeons judiciously adjust to optimize the surgical environment while always ensuring the highest level of safety.

## 4. Temperature and Humidity

Maintaining proper temperature control is essential in surgical procedures as the natural thermoregulation of patients is compromised by the effects of general anesthesia since all hypnotic agents, both intravenous and inhaled, severely disrupt body thermoregulatory function by lowering the point at which defense responses are activated and inhibit vasoconstricting and shivering mechanisms [73]. Intraoperative hypothermia has been linked to several potentially injurious effects. It can alter the hemostatic cascade, since it hampers platelet and clotting factor function [74], thus increasing blood loss and ultimately increasing the need for allogeneic blood transfusion [75]. It can also cause myocardial dysfunction, hypokalemia, and arrythmias [76]. Moreover, it delays both postoperative recovery [77] and wound healing and increases surgical infections and anesthetic drugs’ half-life [78,79,80]. The CO_2_ for pneumoperitoneum is administered dry and at room temperature and body heat is needed to warm and humidify it to physiologic values. Most dissipated energy is spent to humidify the dry gas rather than warm it. Using standard physical constants, it has been calculated that four times the amount of calories is needed to humidify the same volume of dry gas than warm it from 20 °C to 37 °C [81]. The extent to which this phenomenon might substantially reduce core body temperature remains a topic of debate [82]. Early animal data showed that heating the insufflated CO_2_ resulted in higher core and peritoneal temperature in a porcine laparoscopic model compared to standard cold insufflated CO_2_ with a simulated leak of 10 L min^−1^ [83]. These results were confirmed in a rat model where animals undergoing laparoscopy with heated and humidified CO_2_ showed higher intraperitoneal and core temperatures than dry or warm, or humidified only insufflated gas [84], resulting in higher core body temperatures [85]. However, these results were not confirmed in a trial carried out in obese patients undergoing laparoscopic gastric bypass where no difference in core temperature was observed between dry and warm and humidified insufflation [86]. Indeed, a recent systematic review only showed slightly higher core temperatures in patients who underwent laparoscopic surgery with warmed and humidified gas compared to the standard dry and cold gas [87], while another showed a slightly higher core temperature with heated and humidified gas compared to a heated-only gas [88]. These conflicting results can probably result in how much and how long the gas is insufflated and the employed alternative warming methods, such as warming blankets and fluid heaters [89]. One recent observational study conducted in a variety of laparoscopic procedures that ranged from appendectomies to colorectal surgery and hernia repair examined the effect of dry and cold insufflation on core body and peritoneal temperature in patients actively warmed by whole body warm air blanket up to 60 min after surgical incision. They observed how core body temperature was unaffected throughout the measurement period while its difference with intraperitoneal temperature increased significantly [90].

Regardless of the systemic consequences, the effect of cold and dry CO_2_ insufflation on the peritoneal microenvironment should be considered [16]. Animal studies’ data shows that the previously discussed peritoneal injury [39,91,92,93] can be attenuated by humidification. For instance, in an animal RCT comparing the administration of cold and dry versus warm versus humidified insufflated gas, the authors found increased peritoneal injury by electronic microscopy examination and increased intra-abdominal adhesions through direct peritoneal examination two weeks after the laparoscopic intervention [94]. Other authors studying the effect of cold and dry insufflation in mice undergoing laparoscopic surgery observed how abdominal adhesions formation was closely linked to the use of non-humidified gas [95]. There is, however, limited human experimental data regarding peritoneal ultrastructure and the conditions of intraperitoneal temperature and humidity [96]. In an observational study carried out in patients undergoing laparoscopic cholecystectomies, peritoneal biopsies were obtained at the beginning and at the end of the procedure, and the authors report that mesothelial edema, apical cell membrane deterioration, and cell apoptosis can be observed in the latter sample [38]. One contributing factor to this scarcity of data may be the absence of a specialized monitoring system capable of continuously evaluating intra-abdominal temperature and humidity throughout laparoscopy, providing quantitative data since core temperature might not necessarily be an accurate estimation of intraperitoneal temperature. Several commercial devices can humidify and warm the insufflated gas, but, to our knowledge, no intracavitary monitoring system can currently be used as a feedback loop to achieve individualized peritoneal conditioning. Also, the association of the observed peritoneal injury and adhesion formation on clinical outcomes remains to be elucidated.

During laparoscopic procedures, the maintenance of an optimal vision field is key to carrying out the procedure safely and to improve surgical precision and ultimately reduce operative time. One additional benefit of warming and humidifying the insufflated gas is providing a better optical field to the surgeon as laparoscopic lens fogging (LLF) resulting from water condensation can hinder visibility. LLF can also be related to particulate debris, blood, and smoke accumulation on the scope lens [97], and it can increase the surgical time while interfering with the most critical tasks, such as structures’ close-up view, bleeding control, or when there is no haptic feedback, such as in some instances of robotic-assisted surgery. LLF can have various predisposing factors [13,97], although the most accepted theories of its origin revolve around the dew point and psychrometric parameters, i.e., the temperature and humidity of the scope and the surrounding environment. The dew point is the temperature at which condensation happens on a surface, given a specific humidity level and the temperature of the surrounding gas. It has been estimated that to ensure clear vision during laparoscopy, the ambient temperature needs to be above 34 °C when the humidity is around 85%, which is a value that lies to the lower tail of normal laparoscopy. Also, if the temperature of the scope lens exceeds 37.8 °C, there will be no condensation even at 100% humidity. Therefore, it is recommended to keep the scope lens consistently above 37.8 °C to effectively prevent lens fogging [98,99]. Data from animal studies suggest that cooling by convection from the cold insufflation gas caused by the proximity of the injection port to the scope can produce condensation even if it has been pre-warmed [100]. Moreover, if the temperature of the cavity drops below its dew point, fine water droplets in the peritoneal environment can condense into a mist that hinders visibility. This has been related to cold gas insufflation as well [101]. Although these LLF explanations rely on scientific theory and experimental data, extensive comparative analyses on methods of LLF management are still lacking. Indeed, there are even studies that challenge this paradigm [102,103]. All in all, it seems reasonable that administering a warm and humidified gas, especially if accompanied by a supplementary flow, can be beneficial to prevent LLF and, more importantly, preserve peritoneal homeostasis. Indeed, recently developed insufflating systems such as the AirSeal^®^ (ConMed, New York, US) implement an augmented flow to produce gas recirculation to help smoke evacuation and provide a constant clear surgical field that could benefit from additional warmth and humidification [104]. Indeed, in a recent study, the use of this device improved the rate of surgeries that could be carried out at low IAP compared to standard insufflation administration, [105] therefore improving surgeons’ comfort, which can be at times reduced at low IAP [106].

## 5. Conclusions: Perioperative Goals and Implementation

The introduction of laparoscopic surgery has been one of the greatest advances in perioperative care in the last decades. The enhancement of the insufflation procedure can provide potential benefits to couple with the already implemented advancements in vision and precision. In this review, we revise the existing evidence concerning strategies for maintaining peritoneal homeostasis and averting the systemic and clinical ramifications associated with compromised perfusion and mesothelial cell injury. This can be achieved by (1) an individualized and dynamic adjustment of the intraabdominal pressure, (2) applying measures to increase surgical workspace, and (3) regulating the temperature and humidity of the insufflated gas. Future research should focus on validating this approach further, and insufflators should allow for monitoring and control of IAP, volume, temperature, and humidity.

## Data Availability

The paper does not report any original data.
